# Prevalence and Risk Factors of Endometrial Carcinoma Associated with Endometrial Polyps: A Retrospective Study of 2588 Polish Patients

**DOI:** 10.3390/diagnostics16172821

**Published:** 2026-09-02

**Authors:** Zofia Maria Kiestrzyn, Maciej Wilczak, Karolina Chmaj-Wierzchowska

**Affiliations:** 1 Medical Faculty, Poznan University of Medical Sciences, 10 Fredry Street, 61-701 Poznan, Poland; 2 Student Scientific Society of Obstetrics and Gynecology, Poznan University of Medical Sciences, 10 Fredry Street, 61-701 Poznan, Poland; 3 Department of Maternal and Child Health and Minimally Invasive Gynecological Surgery, Poznan University of Medical Sciences, 33 Polna Street, 60-535 Poznan, Poland; mwil@ump.edu.pl; 4 Laboratory of Modern Interventional Therapies in Gynecology and Urogynecology, Poznan University of Medical Sciences, 33 Polna Street, 60-535 Poznan, Poland

**Keywords:** endometrial polyp, endometrial carcinoma, hysteroscopy under local anesthesia, risk factors, age

## Abstract

**Background/Objectives:** Endometrial polyps are a prevalent pathological condition within the uterine cavity. While the majority of lesions are classified as benign, histopathological examinations indicate a potential for malignant transformation occurring in 0.5–5% of cases. The present study aimed to evaluate the prevalence of endometrial carcinoma and pre-malignant histopathological findings (glandular hyperplasia without atypia and atypical glandular hyperplasia) associated with endometrial polyps and to identify pertinent risk factors among Polish women undergoing hysteroscopic polypectomy under local anesthesia. **Methods:** A retrospective cohort study was conducted at the Outpatient Hysteroscopy Center of the Heliodor Swiecicki University Hospital of Gynecology and Obstetrics, affiliated with Poznan University of Medical Sciences. The study included patients treated using the GUBBINI mini-resectoscope under local anesthesia with the Hystero-Block system (Tontarra Medizintechnik GmbH, Wurmlingen, Germany) between December 2022 and July 2026. Statistical analysis of risk factors was performed using the Kruskal–Wallis H test and Fisher’s exact test. Odds ratios for potential risk factors were also calculated. **Results:** The study comprised 2588 patients. Endometrial carcinoma associated with endometrial polyps was identified in 16 women (0.62%). Additional histopathological findings included glandular hyperplasia in 360 patients (13.91%) and atypical hyperplasia in 35 patients (1.35%), with the remaining patients diagnosed with benign uterine polyps (84.12%). Patients with malignant and pre-malignant lesions were significantly older than those with benign polyps (*p* < 0.001). Regarding polyp size, patients diagnosed with glandular hyperplasia without atypia had significantly larger polyps than those with benign lesions (*p* < 0.001). Estimated postmenopausal status was associated with significantly higher odds of endometrial carcinoma (Odds Ratio [OR] = 6.56, 95% Confidence Interval [CI]: 2.12–20.30, *p* = 0.001) and atypical glandular hyperplasia (OR = 3.13, 95% CI: 1.41–6.96, *p* = 0.005) compared to benign polyps. Similarly, obesity increased the odds of glandular hyperplasia without atypia (OR = 1.32, 95% CI: 1.01–1.74, *p* = 0.045) and atypical glandular hyperplasia (OR = 3.33, 95% CI: 1.67–6.65, *p* < 0.001). Furthermore, hypertension was associated with higher odds of glandular hyperplasia without atypia compared to benign polyps (OR = 1.41, 95% CI: 1.02–1.97, *p* = 0.040). Other evaluated risk factors, including type II diabetes mellitus, infertility, abnormal uterine bleeding, breast cancer history, hypothyroidism and polyendocrine metabolic ovarian syndrome (PMOS), did not reach statistical significance (*p* > 0.05). This large cohort provides important data regarding the prevalence and risk factors of carcinoma associated with endometrial polyps and pre-malignant uterine lesions in Polish women. **Conclusions:** Endometrial carcinoma associated with endometrial polyps is a rare entity among Polish women undergoing outpatient hysteroscopy (0.62%). Nevertheless, the primary diagnostic value of this large-scale study lies in demonstrating that despite the low prevalence of malignancy in standard “see-and-treat” outpatient settings, systematic histopathological evaluation remains an absolute diagnostic mandate, as relying solely on visual or clinical parameters is insufficient to exclude malignancy. Furthermore, identified risk factors, such as age, polyp size, estimated postmenopausal status, obesity and hypertension, serve as critical diagnostic red flags, associated with increased odds of concurrent premalignant lesions or carcinoma.

## 1. Introduction

Endometrial polyps (EPs) are localized overgrowths of endometrial glands, stroma, and blood vessels protruding into the uterine cavity [1]. They are considered the most common intrauterine lesions, with a reported prevalence ranging from 7.8% to 34.9% [2]. The occurrence of EPs is associated with several factors, including age, hormonal disturbances, obesity, infertility, polyendocrine metabolic ovarian syndrome (PMOS), late menopause, and the use of tamoxifen or hormone replacement therapy (HRT) [3,4].

EPs may occur in women of all age groups; however, they are most frequently diagnosed between 40 and 49 years of age [5]. The prevalence of EPs is higher in postmenopausal women than in premenopausal women [2]. Although most EPs are benign lesions, malignant or premalignant lesions may coexist within polyps in approximately 0.5–5% of cases [2,6,7]. The risk appears to increase in postmenopausal women and in patients presenting with abnormal uterine bleeding (AUB) [7,8,9]. Additional reported risk factors include obesity, hypertension, and diabetes mellitus [6].

Because EPs are frequently asymptomatic, they are commonly detected during routine transvaginal ultrasonography, particularly with Power Doppler imaging [2,4]. The most common clinical symptom associated with EPs is AUB, with EPs identified in approximately 10–40% of women presenting with abnormal bleeding [6].

Endometrial carcinoma remains one of the most common gynecological malignancies worldwide. In Poland, endometrial carcinoma accounts for approximately 7% of all malignancies diagnosed in women [10]. Despite its relatively favorable prognosis when diagnosed early, delayed diagnosis may occur because of the asymptomatic nature of early-stage disease.

According to current international guidelines, hysteroscopy with targeted biopsy represents the gold standard for the diagnosis and treatment of EPs [11,12,13]. Hysteroscopic polypectomy enables complete lesion removal and histopathological assessment, which is essential for excluding malignant transformation.

The present study aimed to evaluate the prevalence of endometrial carcinoma and pre-malignant histopathological findings (glandular hyperplasia without atypia and atypical glandular hyperplasia) associated with EPs and to explore potential risk factors among Polish women undergoing hysteroscopic polypectomy at a tertiary university referral center between December 2022 and July 2026.

## 2. Materials and Methods

### 2.1. Study Design and Setting

This retrospective cohort study was conducted at the Department of Maternal and Child Health and Minimally Invasive Gynecological Surgery, Poznan University of Medical Sciences, Poland. The study included patients admitted to the Outpatient Hysteroscopy Center of the Heliodor Swiecicki University Hospital of Gynecology and Obstetrics, Karol Marcinkowski University of Medical Sciences in Poznan, for hysteroscopic procedures performed under local anesthesia (paracervical anesthesia using lidocaine) between December 2022 and July 2026.

### 2.2. Study Group

**Figure 1:**
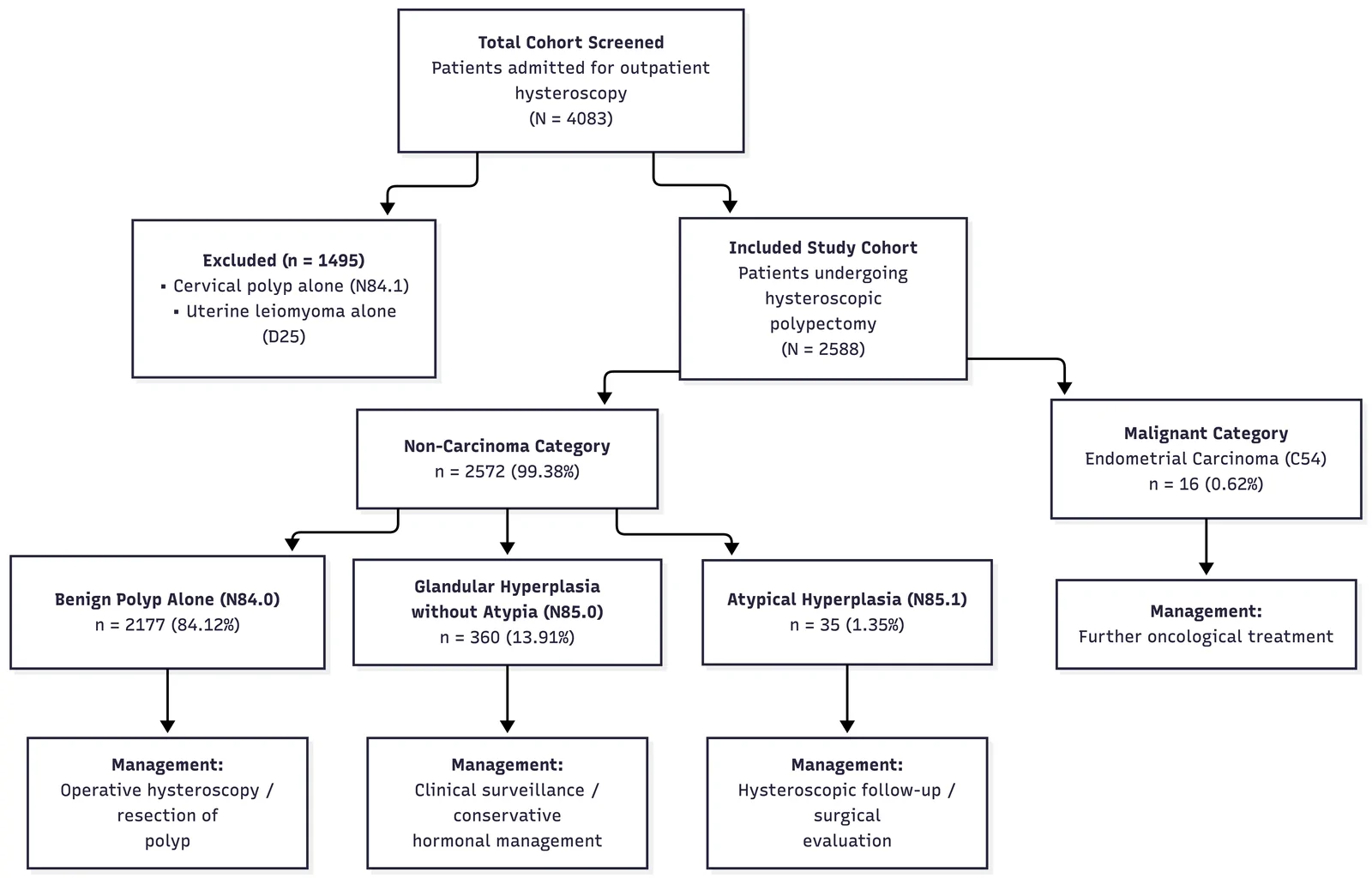
Inclusion and exclusion criteria, classification, number of patients, and patient management based on histopathological diagnosis.

The medical records of 4083 patients were retrospectively analyzed, and a total of 2588 patients who underwent hysteroscopic polypectomy were identified for the study. Histopathological examination revealed endometrial carcinoma associated with EPs in 16 patients, glandular hyperplasia without atypia in 360 patients, atypical glandular hyperplasia in 35 patients, and benign polyps in 2177 patients. No patient was diagnosed with endometrial carcinoma in situ (Figure 1).

Patients were eligible if they underwent hysteroscopic resection of at least one endometrial polyp and had an available histopathological diagnosis. Histopathological findings were subsequently classified as non-carcinoma or malignant according to the corresponding ICD-10 codes. The following inclusion criteria were applied: hysteroscopic diagnosis of at least one EP; histopathological confirmation of a benign lesion (endometrial polyp N84.0*, endometrial glandular hyperplasia N85.0*, or endometrial atypical hyperplasia N85.1*); and histopathological confirmation of a malignant lesion (endometrial carcinoma in situ D07.0* or malignant neoplasm of the uterine body C54*). The exclusion criteria were as follows: diagnosis of cervical polyp alone (N84.1*) and diagnosis of uterine leiomyoma alone (D25*).

* ICD-10 classification codes.

### 2.3. Procedural Technique

EPs were hysteroscopically resected using the Hystero-Block system with a GUBBINI mini-resectoscope (Tontarra Medizintechnik GMBH, Wurmlingen, Germany) under local anesthesia. In this approach, thin operative hysteroscopes with a diameter of 5 to 9 mm were used. A normal saline solution was used as the distension medium, and polypectomy was performed with electrodes based on a “see-and-treat” technique. The Hystero-Block system minimizes patient pain. The procedure usually lasted for 7–12 min. Patients were discharged from the outpatient unit approximately 120 min after completion of the procedure.

### 2.4. Statistical Analysis and Language Editing

Statistical analysis was performed using Statistica 13.0 and Jamovi 2.8.2. Age and polyp size were non-normally distributed and were compared using the Kruskal–Wallis H test with the Dwass–Steel–Critchlow–Fligner (DSCF) pairwise comparison, if applicable. Categorical variables were evaluated using Fisher’s exact test. Because only 16 malignant events were observed, no multivariable model was fitted. Exploratory univariable odds ratios (OR) with 95% confidence intervals (CI) were calculated for *N* = 2111 using the multinomial logistic regression, with benign polyp group as the reference level; factors with 0 cell count in any classification group were excluded from the OR calculation. A two-sided *p*-value < 0.05 was considered statistically significant. Clinical chart completeness differed by variable. During the preparation of this manuscript, the authors used ChatGPT (OpenAI, GPT-5.3) to assist with language editing and improvement of readability.

## 3. Results

Among the 2588 patients included in the analysis, histopathological examination identified endometrial carcinoma associated with an endometrial polyp in 16 patients (0.62%). A total of 2177 patients had a benign polyp (84.12%); glandular hyperplasia without atypia was identified in 360 patients (13.91%) and atypical glandular hyperplasia in 35 patients (1.35%). No carcinoma in situ was identified (Table 1).

As shown in Table 1, both age and polyp size differed significantly between the four histopathological groups (Kruskal–Wallis *p* < 0.001 for both). Post hoc analysis using the DSCF test revealed multiple specific group differences. Patients with benign polyps had a median age of 42.0 years (Interquartile range [IQR] 15.0) and were significantly younger than those with endometrial carcinoma (median 56.5, IQR 17.8, *p* = 0.018), hyperplasia without atypia (median 45.0, IQR 15.0, *p* < 0.001), and atypical hyperplasia (median 56.0, IQR 17.5, *p* < 0.001). Additionally, patients with atypical hyperplasia were significantly older than those with hyperplasia without atypia (*p* = 0.001). Regarding polyp size, the DSCF test indicated that polyps exhibiting hyperplasia without atypia (median 10.0, IQR 7.00) were significantly different in size (*p* < 0.001) compared to benign polyps (median 10.00, IQR 8.0); atypical hyperplasia and carcinoma groups recorded medians of 10.0 mm (IQR 9.00) and 15.0 mm (IQR 6.00), respectively, with no other pairwise comparisons reaching statistical significance.

As presented in Table 2, the results for bivariate analysis of potential clinical risk factors across the four histopathological groups revealed statistically significant differences for estimated postmenopausal status (*p* < 0.001), hypertension (*p* = 0.002), and obesity (*p* = 0.002). Estimated postmenopausal status was most frequently observed in the carcinoma (68.8%) and atypical glandular hyperplasia (54.3%) groups, compared to benign polyps (23.4%). Similarly, the prevalence of hypertension and obesity increased progressively across premalignant and malignant subgroups, reaching 37.5% and 41.2% in the atypical hyperplasia group, respectively. Conversely, other clinical comorbidities including AUB, Type II diabetes mellitus, infertility, breast cancer, hypothyroidism and PMOS, did not show statistical significance with histopathological classification (*p* > 0.05).

**Table 2:** Distribution of clinical characteristics and potential risk factors across four histopathological subgroups of endometrial polyps.

Risk Factor	Carcinoma (*n* = 16) *	Benign Polyp (*n* = 2177) *	Glandular Hyperplasia Without Atypia (*n* = 360) *	Atypical Glandular Hyperplasia (*n* = 35) *	*p*-Value (α = 0.05) ^5^
Estimated postmenopausal status ^1^	11 (68.8%)	510 (23.4%)	102 (28.3%)	19 (54.3%)	**<0.001**
Hypertension ^2^	2 (13.3%)	288 (16.4%)	69 (22.5%)	12 (37.5%)	**0.002**
AUB	0 (0.00%)	110 (5.05%)	17 (4.72%)	3 (8.57%)	0.655
Obesity ^3^	4 (25%)	375 (17.4%)	78 (21.8%)	14 (41.2%)	**0.002**
Type II diabetes mellitus ^2^	2 (13.3%)	63 (3.58%)	12 (3.92%)	3 (9.38%)	0.065
Infertility ^2^	0 (0.00%)	4 (0.23%)	0 (0.00%)	0 (0.00%)	1.000
Breast cancer ^2^	0 (0.00%)	40 (2.28%)	6 (1.96%)	0 (0.00%)	1.000
Hypothyroidism ^2^	3 (20.0%)	362 (20.6%)	57 (18.6%)	5 (15.6%)	0.818
PMOS ^4^	0 (0.00%)	0 (0.00%)	1 (0.39%)	0 (0.00%)	0.169

* Sample sizes for specific variables varied slightly due to missing clinical chart data. ^1^ Menopausal status was unavailable for 607 patients. For these patients, age ≥ 50 years was used as a pragmatic proxy for postmenopausal status. Therefore, this variable is reported as estimated postmenopausal status. ^2^ Percentage values calculated for *N* = 2111: carcinoma (*n* = 15), benign polyp (*n* = 1758), glandular hyperplasia without atypia (*n* = 306), atypical glandular hyperplasia (*n* = 32). ^3^ Percentage values calculated for *n* = 2564: carcinoma (*n* = 16), benign polyp (*n* = 2156), glandular hyperplasia without atypia (*n* = 358), atypical glandular hyperplasia (*n* = 34). ^4^ Percentage values calculated for *N* = 1789: carcinoma (*n* = 15), benign polyp (*n* = 1487), glandular hyperplasia without atypia (*n* = 257), atypical glandular hyperplasia (*n* = 30). ^5^ Statistically significant associations (*p* < 0.05) are **bolded**.

Given the small sample size of the study group (*n* = 16), definitive conclusions regarding the impact of tamoxifen use (related to breast cancer history) or hormone therapy on endometrial carcinoma development could not be derived.

Multinomial logistic regression analysis using the benign polyp as the reference category (*N* = 2111) identified several independent predictors of endometrial pathology (Table 3). Estimated postmenopausal status was associated with significantly increased odds of atypical glandular hyperplasia (OR = 3.13, 95% CI: 1.41–6.96, *p* = 0.005) and endometrial carcinoma (OR = 6.56, 95% CI: 2.12–20.30, *p* = 0.001). Obesity also independently elevated the likelihood of glandular hyperplasia without atypia (OR = 1.32, 95% CI: 1.01–1.74, *p* = 0.045) and atypical glandular hyperplasia (OR = 3.33, 95% CI: 1.67–6.65, *p* < 0.001). Furthermore, hypertension was significantly associated with higher odds of glandular hyperplasia without atypia (OR = 1.41, 95% CI: 1.02–1.97, *p* = 0.040).

Other evaluated comorbidities, including Type II diabetes mellitus and hypothyroidism, did not show statistically significant associations across the pathological subgroups (*p* > 0.05) (Table 3).

## 4. Discussion

EPs are the most common pathology encountered in gynecological practice. Their asymptomatic nature often leads to incidental detection during routine gynecological ultrasound assessments. The reported risk of malignant transformation within an EP into endometrial carcinoma varies widely in the literature, typically ranging from 0.5% to 5.0%, with some high-risk cohorts reporting rates up to 7.2% [7,14].

In the present large-scale cohort of 2588 women undergoing outpatient hysteroscopic polypectomy under local anesthesia, the observed prevalence of EP-associated endometrial carcinoma was 0.62% (*n* = 16). This value lies at the lower end of the range reported in previous international studies. Because of the large study population (*n* = 2588), the observed proportion of patients with EP-associated endometrial carcinoma among Polish women could be considered a significant contribution to the current literature. The low malignancy rate observed in this study must be interpreted in the context of the outpatient “see-and-treat” setting. Office hysteroscopy under local anesthesia is a rapid, minimally invasive approach that permits direct visualization, targeted treatment, and histopathological assessment [2,15]. Because this cohort was derived from an outpatient pathway, patients with severe obesity, major comorbidities, technically complex lesions, or highly suspicious findings may have been preferentially referred to an operating-room pathway under general anesthesia. This referral pattern may have introduced selection bias and may partly explain the relatively low prevalence of carcinoma observed in this series. Nonetheless, the identification of 16 carcinomas alongside 35 cases of atypical glandular hyperplasia (1.35%) and 360 cases of glandular hyperplasia without atypia (13.91%) emphasizes that macroscopic hysteroscopic appearance alone cannot reliably rule out malignancy. Histopathological assessment of hysteroscopically removed polyps remains essential for definitive diagnosis and for identifying malignant or premalignant lesions, even in low-risk ambulatory cohorts [16].

In contrast to some studies suggesting an association between a larger polyp size and an increased risk of malignancy, no such significant relationship was observed in the present study [17,18]. In the present cohort, polyp size was not significantly associated with carcinoma (*p* > 0.05). This finding should be interpreted cautiously because polyp-size data were available for only 11 of 16 carcinoma cases, limiting the statistical power. However, the DSCF test indicated that polyps exhibiting hyperplasia without atypia were significantly larger in size compared to benign polyps (*p* < 0.001). Cetin et al. demonstrated that polyp length and endometrial thickness were significantly higher in the malignant lesion group than in the benign lesion group. However, the groups showed no significant differences in age, body mass index, number of pregnancies and deliveries, number of miscarriages, education level, smoking status, surgical history, comorbidities, or symptoms [18].

Age is another variable that differed significantly between the four histopathological groups (*p* < 0.001). More specifically, patients diagnosed with carcinoma (median 56.5 years) and atypical hyperplasia (median 56.0 years) were significantly older than those with benign polyps (median 42.0 years; *p* = 0.018 and *p* < 0.001, respectively). On univariable multinomial logistic regression, estimated postmenopausal status conferred a more than sixfold increase in the odds of carcinoma (OR= 6.56, 95% CI: 2.12–20.30, *p* = 0.001) and a threefold increase in atypical glandular hyperplasia (OR = 3.13, 95% CI: 1.41–6.96, *p* = 0.005) compared to benign polyps. Although postmenopausal status was estimated using age >50 years for the 607 (23.5%) records lacking direct menstrual cycle records, this pragmatic proxy remains clinically grounded and aligns with standard epidemiological practice [19,20,21,22]. This approach may misclassify individual women; therefore, this variable should be interpreted as estimated rather than directly observed menopausal status.

Additional metabolic risk factors for malignant transformation include obesity, hypertension, and diabetes mellitus [7,9,14,18]. In the present study, the malignant group showed higher prevalence of obesity and diabetes mellitus type II compared to the benign polyp group; however, this difference was not statistically significant due to the malignant group’s small size (*n* = 16) and missing data on comorbidities. These findings are partially inconsistent with previous reports highlighting the role of metabolic syndrome and hormonal disturbances in the pathogenesis of endometrial carcinoma [23,24]. Nevertheless, the lack of statistical significance observed in the present study could be attributed to the limited number of patients with confirmed malignancy.

However, metabolic factors demonstrated subgroup-specific associations for hyperplasia without atypia and atypical hyperplasia. Obesity was significantly associated with elevated odds of glandular hyperplasia without atypia (OR = 1.32, 95% CI: 1.01–1.74, *p* = 0.045) and atypical glandular hyperplasia (OR = 3.33, 95% CI: 1.67–6.65, *p* < 0.001), while hypertension increased the odds of hyperplasia without atypia (OR = 1.41, 95% CI: 1.02–1.97, *p* = 0.040).

Moreover, the impact of tamoxifen use and HRT could not be reliably assessed in the present study. As reported earlier, tamoxifen may increase the risk of development of endometrial proliferative lesions, including polyps and carcinoma, particularly with long-term use [25]. In contrast, the effect of HRT on the development of malignant lesions depends on its regimen and composition [23].

When carcinoma is identified within an endometrial polyp, subsequent management requires complete oncological assessment and staging according to current endometrial cancer guidelines [26]. Because this study did not collect data on peritoneal dissemination, definitive FIGO stage, lymph-node status, or oncological outcomes, no conclusions regarding these issues can be drawn from this cohort.

## 5. Study Limitations

Several limitations of this study should be acknowledged. First, the retrospective and single-center design inherently limits the ability to establish causal relationships and may reduce generalizability of the findings to other populations and healthcare settings. Although the relatively large overall cohort (*N* = 2588) strengthens the descriptive value of the study, the number of patients diagnosed with endometrial carcinoma was small (*n* = 16). Consequently, multivariable adjustment was not feasible, and the analyses were restricted to univariable models, resulting in reduced statistical power and wide confidence intervals. Furthermore, zero event counts in certain clinical categories (such as AUB, breast cancer history, and infertility) led to complete separation, precluding regression estimation for these factors. The results of the risk-factor analyses should therefore be interpreted cautiously and regarded as exploratory.

Second, clinical chart completeness varied across parameters. In particular, polyp size was documented for 11 of the 16 carcinoma cases, limiting the statistical power to identify dimensional cutoff thresholds. Additionally, direct menopausal status was unavailable for 607 patients; while using age > 50 as standard pragmatic proxy, this approach might introduce non-differential misclassification. Finally, the retrospective nature of data collection precluded standardized assessment of all potentially relevant risk factors, including detailed hormonal exposure, tamoxifen use, and family history of endometrial malignancy, or other metabolic or reproductive factors.

Third, the study population consisted of women undergoing hysteroscopic procedures predominantly in an outpatient setting under local anesthesia. Patient selection for this setting may have introduced selection bias. Women with severe obesity, substantial medical comorbidities, technically challenging intrauterine pathology, or large or highly suspicious lesions may have been preferentially referred for procedures in the operating room under general anesthesia rather than managed in the outpatient setting. Similar selection according to lesion characteristics and the anticipated complexity of hysteroscopic treatment has been described in previous studies of office hysteroscopy. Therefore, the present cohort may underrepresent patients with more complex disease or a higher pre-procedural suspicion of malignancy, potentially affecting the observed prevalence of endometrial carcinoma and limiting the generalizability of our findings to all women with endometrial polyps.

Finally, the study reflects clinical practice at a tertiary center using a specific hysteroscopic technique and experienced operators. Differences in patient selection, equipment, operator experience, and local referral pathways may influence outcomes in other institutions. Future prospective, multicenter studies with standardized data collection and larger numbers of malignant cases are warranted to validate the identified associations and to develop more robust risk-stratification models for malignancy in women with endometrial polyps.

## 6. Conclusions

1. Endometrial carcinoma associated with endometrial polyps is a rare entity among Polish women undergoing outpatient hysteroscopy (0.62%). Nevertheless, the primary diagnostic value of this large-scale study lies in demonstrating that despite the low prevalence of malignancy in standard “see-and-treat” outpatient settings, systematic histopathological evaluation remains an absolute diagnostic mandate, as relying solely on visual or clinical parameters is insufficient to exclude malignancy. Furthermore, identified risk factors, such as age, polyp size, estimated postmenopausal status, obesity and hypertension, serve as critical diagnostic red flags, associated with increased odds of concurrent premalignant lesions or carcinoma.
2. Estimated postmenopausal status was the strongest independent risk factor, conferring over sixfold higher odds of carcinoma (OR = 6.56) and threefold higher odds of atypical glandular hyperplasia (OR = 3.13) compared to benign polyps.
3. Metabolic comorbidities, particularly obesity and hypertension, were significantly associated with increased odds of premalignant endometrial hyperplasia but did not reach statistical significance for carcinoma alone, likely constrained by the small number of malignant events (*n* = 16). Consequently, risk-factor estimates should be interpreted cautiously as exploratory and warrant validation in larger multicenter cohorts.

## Figures and Tables

**Table 1:** Clinical characteristics and continuous variables of patients within four histopathological groups (*n* = 2588).

Variable Median (IQR)	Carcinoma Group (*n* = 16; 0.62%)	Benign Polyp Group (*n* = 2177; 84.12%)	Glandular Hyperplasia Without Atypia (*n* = 360; 13.91%)	Atypical Glandular Hyperplasia (*n* = 35; 1.35%)	***p*** **-Value** **(α = 0.05) ^2^**
Age (years)	56.5 (17.8)	42.0 (15.0)	45.0 (15.0)	56.0 (17.5)	**<0.001**
Polyp size (mm) ^1^	15.0 (6.00)	10.0 (8.00)	10.0 (7.00)	10.0 (9.00)	**<0.001**

IQR, interquartile range. ^1^ Polyp-size data was available for 11 patients in the carcinoma group, 1739 patients in the benign polyp group, 313 patients in the glandular hyperplasia without atypia group, and for 27 patients in the atypical glandular hyperplasia group. ^2^ Statistically significant associations (*p* < 0.05) are **bolded**.

**Table 3:** Results for multinomial logistic regression analysis of potential risk factors associated with premalignant and malignant endometrial pathology compared to benign polyps ^1,2,3^.

Risk Factor	Carcinoma vs. Benign Polyp OR (95% CI), *p*-Value	Glandular Hyperplasia Without Atypia vs. Benign Polyp OR (95% CI), *p*-Value	Atypical Glandular Hyperplasia vs. Benign Polyp OR (95% CI), *p*-Value
Estimated postmenopausal status	**6.56 (2.12–20.30), 0.001**	1.13 (0.839–1.51), 0.429	**3.13 (1.41–6.96), 0.005**
Hypertension	0.293 (0.061–1.40), 0.123	**1.41 (1.02–1.97), 0.040**	1.70 (0.748–3.841), 0.206
AUB ^4^	N/A	N/A	N/A
Obesity	1.58 (0.505–4.92), 0.433	**1.32 (1.01–1.74), 0.045**	**3.33 (1.67–6.65), <0.001**
Type II diabetes mellitus	2.72 (0.554–13.30), 0.218	0.90 (0.468–1.72), 0.744	1.36 (0.384–4.84), 0.631
Infertility ^4^	N/A	N/A	N/A
Breast cancer ^4^	N/A	N/A	N/A
Hypothyroidism	1.03 (0.286–3.73), 0.961	0.90 (0.660–1.23), 0.510	0.79 (0.300–2.08), 0.634
PMOS ^4^	N/A	N/A	N/A

^1^ Models estimated using sample size of *N* = 2111. ^2^ Benign polyp was used as the reference category (OR = 1.00) for all calculations. ^3^ Statistically significant associations (*p* < 0.05) are **bolded**. ^4^ Variables with a zero cell count in any histopathological classification group were excluded from this analysis.

## Data Availability

The data presented in this study are available on request from the corresponding author.
